# Informing the design of a national screening and treatment programme for chronic viral hepatitis in primary care: qualitative study of at-risk immigrant communities and healthcare professionals

**DOI:** 10.1186/s12913-015-0746-y

**Published:** 2015-03-13

**Authors:** Lorna Sweeney, John A Owiti, Andrew Beharry, Kamaldeep Bhui, Jessica Gomes, Graham R Foster, Trisha Greenhalgh

**Affiliations:** Institute for Health and Human Development, University of East London, UH250, Stratford Campus, Water Lane, London, E15 4LZ UK; Centre for Psychiatry, Wolfson Institute of Preventive Medicine, Barts and The London School of Medicine and Dentistry, Queen Mary University of London, Charterhouse Square, London, EC1M 6BQ UK; Internal Medicine and Gastroenterology, San Fernando General Hospital, Independence Avenue, San Fernando, Trinidad and Tobago; The Liver Unit, Centre for Digestive Diseases, Blizard Institute, Barts and The London School of Medicine and Dentistry, Queen Mary University of London, 4 Newark Street, London, E1 2AT UK; Nuffield Department of Primary Care Health Sciences, University of Oxford, New Radcliffe House, Radcliffe Observatory Quarter, Woodstock Road, Oxford, OX2 6GG UK

**Keywords:** Screening, Hepatitis B, Hepatitis C, Immigrants, Primary care

## Abstract

**Background:**

Effective strategies are needed to provide screening and treatment for hepatitis B and C to immigrant groups in the UK at high risk of chronic infection. This study aimed to build an understanding of the knowledge, beliefs and attitudes towards these conditions and their management in a range of high-risk minority ethnic communities and health professionals, in order to inform the design of a screening and treatment programme in primary care.

**Methods:**

Qualitative data collection consisted of three sequential phases- (i) semi-structured interviews with key informants *(n = 17)*, (ii) focus groups with people from Chinese, Pakistani, Roma, Somali, and French- and English-speaking African communities *(n = 95)*, and (iii) semi-structured interviews with general practitioners *(n = 6)*. Datasets from each phase were analysed using the Framework method.

**Results:**

Key informants and general practitioners perceived that there was limited knowledge and understanding about hepatitis B and C within high-risk immigrant communities, and that chronic viral hepatitis did not typically feature in community discourses about serious illness. Many focus group participants were confused about the differences between types of viral hepatitis, held misconceptions regarding transmission, and were unaware of the asymptomatic nature of chronic infection. Most welcomed the idea of a screening programme, but key informants and focus group participants also identified numerous practical barriers to engagement with primary care-based screening and treatment; including language and communication difficulties, limited time (due to long working hours), and (for some) low levels of trust and confidence in general practice-based care. General practitioners expressed concerns about the workload implications and sustainability of screening and treating immigrant patients for chronic viral hepatitis in primary care.

**Conclusions:**

Strategies to reduce the burden of chronic viral hepatitis in immigrant communities will need to consider how levels of understanding about hepatitis B and C within these communities, and barriers to accessing healthcare, may affect capacity to engage with screening and treatment. Services may need to work with community groups and language support services to provide information and wider encouragement for screening. Primary care services will need ongoing consultation regarding their support needs to deliver hepatitis screening and treatment programmes.

## Background

The Chief Medical Officer for England and Wales recently highlighted that action is needed to address the growing burden of liver disease, for which undiagnosed hepatitis infection is a major contributory factor. [1] Chronic viral hepatitis, due to either hepatitis B (with or without delta superinfection) or hepatitis C, is a global health concern affecting an estimated 500 million people and causing approximately one million annual deaths, mostly through liver diseases, including cirrhosis and cancer [2]. Vaccination is reducing the prevalence of hepatitis B infection and drugs that control virus replication and reduce morbidity are available [3], which are cost effective and recommended by the National Institute for Health and Care Excellence [4]. Current therapies for hepatitis C are based on poorly tolerated pegylated interferon, but the development of highly effective all oral regimes [5] provides an opportunity to eliminate the virus from many regions of the world. However, modelling studies for hepatitis C have shown that simply providing effective therapies will not substantially reduce the burden of disease; [6] increased access to treatment will be needed to modify the prevalence of cirrhosis and liver cancer. Although similar studies have not been performed for hepatitis B infection there is no reason to believe that similar models will not apply. To impact the global burden of disease from chronic viral hepatitis it will therefore be important to improve rates of diagnosis and treatment in high-risk groups.

Chronic viral hepatitis is common in the developing world, where transmission is typically by mother to foetus, or child to child transmission (e.g. hepatitis B in south-east Asia and sub-Saharan Africa) [7], or by blood borne transmission through contaminated medical equipment (e.g. hepatitis C in Egypt, Pakistan and North Africa) [8,9]. Hepatitis B and C are also moderately prevalent in some Eastern European countries, such as Poland, Romania, the Ukraine and Russia; [10,11] where a variety of transmission routes operate. High-income countries have typically had low rates of chronic viral hepatitis, with infection mostly occurring through sexual transmission in adults (hepatitis B) or via contaminated drug paraphernalia (hepatitis C) [8,12]. However, migration-related chronic viral hepatitis is an emerging public health issue in high-income countries [13]. In the UK, studies have identified significantly higher prevalence rates of hepatitis B and C, and increased mortality from the outcomes of chronic infection, in minority ethnic communities (including Black African, Pakistani and Chinese community samples) compared to the white British population [14-17]. Effective strategies are thus needed to provide hepatitis screening and treatment programmes to immigrant groups in the UK at high risk of chronic infection [18]. The success of a screening programme depends on how well it engages target groups [19], whose health beliefs and perspectives are likely to affect acceptance and uptake*.* Previous research conducted in North America [20,21] and Australia [22] (with Asian communities) and the Netherlands (with the Turkish-Dutch community) [23,24] has suggested low levels of knowledge about hepatitis among high-risk minority populations, which appears to be an important factor in whether hepatitis screening is sought. [25] No research to date has explored lay understandings of hepatitis B and C, or attitudes to screening and treatment, in high-risk immigrant communities in the UK.

In this paper we report the findings of a qualitative study that explored knowledge, perceptions and folk models of hepatitis B and C in a number of high-risk immigrant communities in the UK. The study also explored lay and professional perspectives on a proposed model of targeted screening and treatment provision for hepatitis B and C within primary care services. Findings were used to inform a cluster randomised controlled trial of hepatitis screening and treatment in general practices in London, Bradford and Oxford- the *‘HepFree’* study- which involves GP practices sending screening invitation letters to their patients from at-risk immigrant communities.

## Methods

### Study design

Our study focused on immigrant communities from four regions known to be at high risk of hepatitis B and C: China, Pakistan, Africa and Eastern Europe [2,26]. To maximise the trade-off between depth and breadth, the research was conducted with microcosms of each of these communities in the UK - Pakistani communities in east London and Bradford, the Chinese community in central London, Somali and other African communities in south London, and Eastern European (including Roma) communities in east London. We worked closely with community health and advocacy organisations for the development and implementation of the research.

The study was conducted in three phases: (i) individual interviews with 17 key informants (approximately four per community), (ii) 12 focus groups with 95 people from the target immigrant communities (groups ranged from 6 to 10 participants, with an average group size of 8 people) (iii) individual interviews with six general practitioners (GPs) whose practices included members of the target communities. Interim analysis of the findings from each phase of the research was used to inform data collection in the subsequent phase. All study procedures were approved by the Research Ethics Committee at Queen Mary, University of London (No. QMREC2012/02).

### Phase 1: Key informant interviews

We interviewed people working in key roles within hepatitis treatment services and advocacy in the target communities, in order to gather their views on how social and cultural influences might affect hepatitis screening and treatment [27]*.* All provided written informed consent for participation and audio recording. We interviewed staff members from community health organisations (n = 5), healthcare interpreters within hospital and primary care services (n = 5), specialist hepatitis nurses (n = 3), consultant hepatologists (n = 2), an assistant Imam (n = 1) and a sexual health doctor (n = 1). Recruitment occurred through community organisations, invitations to people already known to the research team, and snowball sampling. A semi-structured interview guide (see Appendix 1) was used, but the interview also provided space for discussion of topics raised spontaneously by the interviewee. All interviews were conducted and transcribed verbatim by LS.

### Phase 2: Focus groups

We explored the health beliefs, knowledge and folk models of hepatitis B and C in community members and their responses to a proposal for hepatitis screening and treatment in primary care. Recruitment was facilitated by community health organisations, who had established trust and support within the local immigrant communities that we hoped to reach. These organisations approached community members about focus group participation and written and verbal information about the study was provided in the preferred language of participants. No difficulties with the recruitment process were reported to the research team, though we acknowledge that some sensitivities may not have been readily shared. Two pilot focus groups were initially conducted with the Pakistani community in east London. Focus groups were then held for Pakistani, Chinese, Somali and Polish-speaking Roma communities. We conducted separate male and female groups for each of these communities, in case any potential discussion of the sexual transmission of viral hepatitis caused discomfort and unease for participants within mixed-gender groups. However, we were advised by the organisation for African communities that mixed-gender groups would be appropriate for the French and English-speaking members of their communities, so we organised the groups in accordance with their advice.

A bilingual staff member from the relevant community organisation facilitated each focus group. They had previous experience with facilitating community discussion or support groups, and were briefed in advance regarding the nature and purpose of the research. Facilitators also assisted with the development of the focus group format and question schedule.

Each focus group participant provided written consent for audio recording of the discussions and for anonymised data to be used for research purposes. Participants completed a brief demographic questionnaire. Focus groups were attended by LS or JO (gender matched to participants), who sat in the corner of the room with a second translator, so that they could note any issues that needed further probing or clarification later in the discussion.

A focus group discussion guide (see Appendix 2) was developed from our review of the literature and refined following the outcomes of the pilot focus groups. Discussion began with broad questions about health problems within the community and knowledge and awareness of hepatitis B and C. Facilitators then provided participants with brief verbal information about these conditions. To gauge perceived acceptability of hepatitis screening and treatment in primary care, structured vignettes were used to invite participants to respond to the hypothetical story of a gender-matched character from their community who had received a letter from his/her GP inviting him/her for hepatitis B and C screening (which reflected the design of the *‘HepFree’* study intervention). The use of fictional vignettes in qualitative research allows participant beliefs regarding appropriate action in the situational context to be explored in greater depth, and is also a less threatening method of introducing a potentially sensitive research topic [28,29], as fears or stigma can be projected onto the fictional character rather than owned as personal.

Following each focus group, LS worked with the bilingual facilitators to translate and transcribe the audio recordings. Word-for-word translation of qualitative research data can obscure the communication of meaning in participants’ contributions [30]. Through working jointly with the facilitators to transcribe the recordings, any confusion concerning the explanation of meaning in words and phrases used by the participants were addressed in the process of producing the transcript. Co-transcription also facilitated initial data interpretation, as it involved discussion of culturally specific issues and the meaning in participants’ accounts.

### Phase 3: General practitioner interviews

Individual telephone interviews were conducted with six GPs (four based in London and two in Bradford) to explore experiences with hepatitis screening and treatment with patients from immigrant communities, and perceived acceptability of the *‘HepFree’* intervention in primary care. The London-based GPs worked in areas with high social disadvantage and ethnic-diversity. One was based in a practice with a large patient base from Pakistan and one worked in a practice that specifically worked with recent immigrants, asylum seekers and refugees. The two Bradford-based GPs worked in inner city Bradford. Both worked in practices with a high proportion of South Asian (mostly Pakistani) patients, and one also worked in an additional practice for asylum seekers and refugees, mostly from African countries. A semi-structured topic guide (Appendix 3) was used for the GP interviews, but the interview provided space for discussion of topics raised spontaneously by informants, who gave verbal consent for audio recording. All interviews were conducted and transcribed verbatim by LS.

### Data analysis

Data were managed in accordance with the data protection policy of Queen Mary University of London (available from authors). The datasets from each phase of the research were analysed separately, using the Framework method [31,32]. In an initial orientation and familiarisation stage, LS reviewed the transcripts in depth, noting key ideas and emerging issues that arose from the original research questions and the issues raised by the participants. TG independently reviewed a sub-set of transcripts. The initial concepts were organised into a preliminary thematic framework on an Excel spreadsheet. We then went through each transcript in turn, adding columns to the spreadsheet to accommodate new themes and shaping the draft framework into analytic categories. The spreadsheet format allowed charting of the range and nature of perspectives on each theme. Emerging findings were presented for discussion at team meetings, which facilitated further interpretation and refinement of themes and concepts. Themes from the key informants, focus group and GP datasets were then compared and contrasted. Ongoing analysis and interpretation were also informed by theoretical perspectives from the literature concerning attitudes to health screening and access to healthcare services for vulnerable groups [33,34]. Community health and advocacy organisations were invited to provide comments and feedback on study findings.

## Results

### Characteristics of sample

Table 1 shows demographic characteristics of the key informants and general practitioners. Table 2 summarises the demographics of the focus group participants. Participants were not asked directly whether they had previously been tested for, or received a diagnosis for hepatitis B or C. During five of the focus group discussions, at least one participant voluntarily informed the group that they had hepatitis B or C, and in most of the focus group discussions at least one participant referred to a family member or friend who had experienced hepatitis infection.

**Table 1:** **Demographics of key informants and general practitioners**

	**Key informants**	**General practitioners**
**Total no. of participants**	17	6
**Mean (SD) age (years)**	47 (9)	52 (14)
**Sex:**		
Male	4	5
Female	13	1
**Ethnicity:**		
African	4	1
Asian/Asian British		
- Chinese	3	-
- Pakistani	2	-
- Indian	-	1
White		
- British	3	3
- Eastern European	4	-
Not stated	1	1

**Table 2:** **Demographics of focus group participants**

**Community**	**Chinese community, London (2 groups)**	**Pakistani communities, London and Bradford (4 groups)**	**Roma communities, London (2 groups)**	**Somali community, London (2 groups)**	**African communities, London (2 groups)**
**Total no. participants**	12	35	15	16	17
**Mean (SD) age (years)**	41 (6)	49 (15)	35 (12)	45 (6)	53 (10)
**Sex:**					
- Male	6	18	8	8	4
- Female	6	17	7	8	13
**Generation immigrant**					
- First generation	12	24	15	16	16
- Years in UK: Mean (SD)	10 (10)	30 (13)	10 (4)	13 (5)	12 (4)
- Second generation	-	11	-	-	1
**Highest level of education**					
- No formal education	-	3	-	1	-
- Primary level or below	2	3	1	3	1
- Secondary level	6	5	14	7	11
- University level	2	3	-	5	1
- Not stated	2	21	-	-	2

The key informant, focus group and GP data were highly convergent, each source tending to affirm and extend findings from the others. The main themes identified were: limited awareness and knowledge of hepatitis B and C (especially lack of awareness about the asymptomatic nature of chronic infection and confusion about modes of transmission); mixed views on the nature and level of stigma associated with these conditions; (broadly) positive attitudes to screening and treatment in principle, but numerous practical barriers to uptake of this in practice; and (for some) lack of confidence in the primary care sector. Capacity issues in primary care were identified from GP interviews. We elaborate on these themes in turn below.

### Limited awareness and knowledge of hepatitis B and C

Key informants and general practitioners perceived that there was very limited knowledge about hepatitis B and C (in terms of the nature of the viruses, their transmission and outcomes) within the target immigrant communities. Key informants for the Chinese and Pakistani communities felt that people with an affected family member were aware of these conditions, but specific knowledge was believed to be low. Key informants for the Eastern European (including Roma) and African communities perceived very little awareness and understanding about hepatitis B and C within these communities.

Most focus group participants had heard of hepatitis B and C, but many felt that they did not know much about these illnesses, and that they were rarely discussed unless a family member was affected.

*“Yes we know about the hepatitis B, C. But we only know the name, we don’t know how it is, how it works, we don’t know.”* (Female, Roma community group)

Key informants and general practitioners felt that many people were unaware of the serious outcomes of untreated chronic hepatitis infection, unless family members had experienced liver cancer or other critical outcomes. Some focus group participants identified that untreated hepatitis B and C infection can lead to cancer or death, but others contrasted hepatitis with “more serious” illnesses such as cancer, diabetes, HIV and TB. Key informants pointed out that hepatitis had not been promoted in health education efforts to the same extent as other illnesses or infectious diseases, with the result that it did not typically feature in community discourses about serious illness.

*“Right now, not many people are relating to hepatitis. What we know in the African communities it is HIV, it is TB, diabetes, hypertension- those are the things that are out there.”* (Key informant for African communities, Sexual health doctor)

Key informants who worked in hepatitis treatment services observed that patients from immigrant communities were often unaware whether their diagnosis was for hepatitis B or C. Focus group participants also indicated uncertainty and confusion about the differences between the types of viral hepatitis. Many believed that hepatitis C was a more harmful infection than hepatitis B, due to an (implied) assumption that the letters represented increasing severity.

*“They say A is OK, B is in the middle and C you’re going to judgement day* [death].*”* (Male, Somali community group)

Several participants also speculated that the different types of hepatitis emerge chronologically, (i.e. B becomes C if left untreated).

### Confusion about mode of transmission and risk of becoming infected

Key informants felt that people in the target communities knew little about how hepatitis B and C are transmitted, or the high risk of infection in their own community. They also pointed out that immigrants who had lived in the UK for many years would be unlikely to perceive themselves to be at risk due to a lack of awareness that hepatitis can be a chronic, asymptomatic infection that they may have acquired at a younger age.

[Described a typical exchange when approaching people for testing at community event] *“Oh there’s nothing wrong with us”. I go, “How do you know? You’re Asian aren’t you? You’re Pakistani Muslim aren’t you?”…“…We’ve not done anything wrong, we don’t do drugs”. I go, “You’ve had your head shaved as a baby, you’ve been circumcised as a baby, how can you say you’ve not got the virus?”* (Key informant for Pakistani community, community support worker)

Some focus group participants correctly mentioned at least one of the main routes of transmission for hepatitis B and C, such as through non-sterilised injection practices, sexual transmission, and transmission from mother to child, and in every focus group contact with infected blood was mentioned as a risk factor. However, participants also suggested that hepatitis B or C can be caused by dirty water, poor sanitation, seafood, fried or buttery foods, alcohol use, and mosquitoes, and transmitted via saliva or through sharing food, cups or utensils.

*“I don’t believe that you can get it that easily, like flu. Because then we would all have it…It has to be through contact of blood or injections.”*

*“…If I drink from a cup and it’s not washed properly and the next person drinks it then that’s how it spreads.”* (Female participants, Somali community group)

Our data suggested that different communities held different explanations for how hepatitis is transmitted within the community. In the Chinese groups, much discussion focused on the shared eating practices of Chinese people; family members and friends typically used their own cooking utensils when diagnosed with hepatitis B. Roma groups talked of transmission through surgery or blood transfusions and referred to people they knew who had been infected in this way. Our informant and focus group data suggested that African communities tend to focus on the sexual transmission of viral hepatitis, which may be due to awareness within these communities that many individuals in sub-Saharan Africa are co-infected with HIV and hepatitis B/C (although the association between transmission modes of these infections is limited in African countries) [35].

### No symptoms, no problem

The words used to refer to hepatitis in the languages of the target communities directly translated to mean ‘jaundice’ or ‘yellow’ , which was a source of confusion and appeared to engender a strong perceived association between hepatitis B/C and visible jaundice. Many focus group participants believed that yellow skin, eyes and nails would be apparent in a person with hepatitis B or C and that this was how the person would know that they had the infection. Others (perhaps confusing hepatitis B/C with hepatitis A) believed that acute physical symptoms, such as vomiting, fever and headaches, would accompany hepatitis B or C infection.

Some focus group participants, particularly those who had personal or family experience of chronic viral hepatitis, were aware that the condition could present with tiredness and a general sense of feeling unwell. Whilst a minority of participants were aware that chronic hepatitis B or C infection may be asymptomatic, many participants expressed surprise and concern when they were informed that hepatitis B and C infection may not be accompanied by symptoms, but can still go on to produce serious complications.

Key informants and focus group participants felt that people typically relied upon the presence of symptoms as an indication of infection and cue to action, and therefore, those in their communities who were not experiencing symptoms of ill health would be unlikely to respond to a hepatitis screening invitation.

*“If they think that it’s something that’s not worth their while, you know, ‘I’ve got nothing wrong with me, I don’t have any liver pains, I’m not jaundiced, why would I have hepatitis B? I’m not going to go for screening’.”* (Key informant for Chinese community, community support worker)

The asymptomatic nature of chronic hepatitis infection was also indicated to reduce its perceived severity. Key informants who worked in hepatitis treatment services reported that lack of symptoms often compromised uptake and adherence to treatment, when patients could not see the purpose of being monitored or taking medication if they felt well. Nevertheless, both key informants and focus group participants generally felt that most people diagnosed with hepatitis B or C within the target communities would be keen to receive treatment, particularly once they became aware of the seriousness of the condition.

### Mixed views on stigma

Our data indicated mixed opinions on the nature and extent of stigma associated with hepatitis B and C infection. Some key informants believed that negative attitudes were more likely in those who associated hepatitis with “dirty” practices like sexual transmission or injection drug use, perhaps in the absence of knowledge about other modes of transmission. Stigma was reported to cause reluctance amongst some hepatitis patients to disclose their diagnosis. Stigma was also attributed to lack of public awareness and knowledge about hepatitis B or C, as people felt uncomfortable telling others about a diagnosis which they could not explain. A few informants also indicated that a diagnosis might carry particular stigma for young people, because it could influence opportunities for marriage.

At the same time, many key informants, focus group participants and general practitioners perceived significantly *less* stigma for hepatitis than for cancer, mental health problems and (in particular) HIV. Key informants and general practitioners reported that people from immigrant communities were more open to discussing and testing for hepatitis than for HIV or other sexually transmitted infections, and were typically less distressed by a hepatitis diagnosis, which was often viewed *“like any other illness”*.

While most focus group participants believed that people within their communities would provide support to family members or friends following a hepatitis diagnosis, some participants also referred to fear of infection and, hence, anxiety around social practices such as eating together, sharing utensils or coming into contact with blood or saliva. Key informants indicated that a hepatitis diagnosis can be particularly difficult for immigrants living in the UK without family support, as they often live in temporary accommodation with lots of other housemates, who may be unsupportive due to fear of infection.

*“..one gentleman, his things were packed because he accidently left in the kitchen his medications and the housemates Googled what he was using these for and they found out that it’s hepatitis drugs and then he came in from work and his things were out in the suitcase.”* (Key informant for Eastern European communities, Hospital interpreter)

In contrast, a few participants felt that there was less fear within their communities about interaction with a person with hepatitis compared to a person with HIV or TB, because the latter infections were perceived to cause more serious consequences.

### Attitudes to screening

Key informants, focus group participants and general practitioners stressed individual differences and variation between people in any community in their responses to screening invitations. Many people were expected to ignore a hepatitis screening invitation letter if they had not previously come across the condition within their family or community, or if they felt well. General practitioners spoke about reluctance of some immigrant patients to attend for health checks or vaccinations, necessitating repeated reminders through letters, telephone and text messages.

There was disagreement on whether a hepatitis screening invitation should state that a particular community is at increased risk of hepatitis, since this may be interpreted as associating the entire community with poverty, being “dirty”, or being from a less civilised or advanced country. An invitation for screening may cause some people to be suspicious about why they had been selected, including perceived targeting by immigration services. Key informants and focus group participants from African communities referred to a suspicion within these communities that blood testing may be used to secretly test for other conditions, or to deliberately infect people with an illness. In this context, any mention of research in the invitation to screening was thought likely to increase suspicion and reduce uptake. Some participants also suggested that people in their communities might fear the next steps (such as invasive scans or other unpleasant tests) in the hepatitis testing process, or the side effects of treatment if they tested positive.

Key informants and focus group participants considered that attending optional preventive health services might not be a priority for many immigrants who were already experiencing significant social and economic pressures. Screening might lead to a person being diagnosed with a serious illness when they did not have the emotional reserves or social support to cope with this.

*“I got a smear test invite for women who are over 40. After I got my invite I said to myself, “What if they diagnose me with this (word meaning ‘horrible thing’)?” So I did not go. And until now I never went. So I’m very scared, it’s very hard for me to go.”* (Female, Somali community group)

Advice from peers was perceived to be an important influence on decision-making about health issues. If a person’s family and friends did not understand hepatitis and its implications, they might discourage attendance for screening. While some key informants, focus group participants and general practitioners believed that a letter from a doctor would be taken seriously and prompt attendance for screening, others felt that in addition to this, more widespread communication about hepatitis and hepatitis screening through community networks and religious organisations would help increase uptake, as would primary care staff raising the idea of hepatitis screening when patients attended for other reasons. General practitioners had observed that people were generally willing to be tested for hepatitis when their doctor had discussed the reasons for screening with them in advance.

Most focus group participants (who may have been a particularly health-conscious sample) said that they themselves would accept the offer of hepatitis screening, and that they would advise their family and friends to respond similarly. These participants viewed screening as a socially responsible activity and a good opportunity to protect oneself from illness and gain peace of mind about one’s health. They suggested that a hepatitis screening programme ought to be framed in this way to their community. However, a number of participants felt that they and others from their community would ‘*ignore’* or *‘avoid’* a screening invitation letter, particularly if it was not compulsory. Taking up a screening invitation was an active choice and involved effort; participants felt that if the person was feeling well and did not perceive a need to be tested, this effort may not be considered worthwhile. Participants also believed that people may ignore the first letter, expecting to be contacted a second time if the screening was important.

*“It depends on the person, some will make an appointment straight away and some, like me, will avoid it and become lazy.”* (Female, Pakistani community group)

Age and gender were potential influences on response to screening invitation. Younger people were perceived as more proactive about their health, more open to testing for sexually transmitted infections, and to have greater access to health information. But they were also viewed as more likely to perceive themselves as healthy and not in need of screening. Women were generally seen as more concerned about health and more likely to be in regular contact with their general practitioner (with the exception of women from traditional Pakistani families), while men generally did not want to make contact with health services unless they were feeling very ill.

### Barriers to uptake of a primary care based service

Key informants felt that while a screening and treatment programme offered through NHS primary care would be popular and convenient for many people, it would not reach at-risk immigrants who lacked legal status in the UK and/or were not registered with a general practice. Focus group participants spoke about the difficulty getting appointments in general practice services and delays between making an appointment and seeing the doctor, perhaps reflecting the significant pressures on the primary care sector. The long working hours and limited employment rights (e.g. lack of formal contracts, no sick pay) of many people from immigrant communities were also viewed as a significant barrier to accessing healthcare services–and hence to uptake of screening and treatment.

*“Especially those in the catering section, they work more than 10 hours a day, six days a week. Even if they are not feeling well they don’t have time to see a doctor. If this person has hepatitis B and there are no symptoms, even if he knows he has it, he doesn’t want to go for a check-up.”* (Male, Chinese community group)

Limited English proficiency was also identified as a significant barrier to accessing screening and treatment for hepatitis. Primary care services were perceived to vary widely in their provision of interpreting services. Patients from the Pakistani communities were often registered with a general practitioner who spoke their language; though because practices had recently become larger in size, with a wider patient base from new immigrant groups, this could not always be accommodated. Many immigrant patients appeared to rely on family members or friends to interpret letters from healthcare services and to communicate on their behalf at medical appointments– an arrangement that can prove difficult if the consultation involves sensitive topics or specialist medical terminology, and which may put pressure on the bilingual relative to take time off work to act as an unpaid translator. Language and communication difficulties prompted some immigrant patients to consult with private general practitioners or traditional medicine practitioners who spoke their language. The provision of screening invitation letters in the languages of the target communities and the provision of interpreting services were widely considered to be essential prerequisites for a successful hepatitis screening and treatment service:

*“I had a friend, I told him that I had hepatitis B. I told him to go for the test because he used to come to my place for meals. He told me ‘How can I go for a test? I don’t speak English’. He said he’s worried about going because he can’t speak English.”* (Female, Chinese community group)

Key informants for the Chinese, Pakistani and Roma communities stressed that a considerable proportion of people from these communities had difficulty reading written information provided in their spoken languages; therefore, information about hepatitis and hepatitis screening would also need to be provided in alternative formats.

Acceptability of primary care based screening and treatment services was seen to depend on the quality of the relationship between the patient and the general practitioner. Key informants and focus group participants believed that such a model would appeal particularly to people who had a longstanding, trusting relationship with a regular general practitioner who understood their health history. Such a situation was by no means universal amongst immigrant communities. New immigrants in particular were sometimes perplexed by primary care services and surprised and disappointed not to receive a referral to secondary care (which would have been standard in their country of origin) when they presented to their general practitioner. Some general practitioners were perceived to *“rush”* appointments and not provide patients with the opportunity to talk through symptoms and explain their concerns, leading to loss of patient confidence:

*“I cut off the relationship with my GP because I never had the chance to express myself about my pain. So now when I’m not feeling well I go to the hospital straight away.”* (Male, French-speaking African group)

Women in the Roma, Somali and Pakistani communities tended to feel more comfortable attending a female general practitioner. Participants in the Roma and Somali community groups described consulting with private doctors in order to have sufficient time for explanations and information about illnesses. Informants for the Eastern European communities indicated that many people from these communities travel back to their home countries for healthcare, for easier access to medical tests and consultations, and because they had greater confidence in the standard of care received (sometimes equated with number of tests ordered or referrals to specialists). Key informants and general practitioners observed that many immigrant patients prefer to consult with specialist doctors for the treatment of illness and thus may feel *“fobbed off”* by primary care based treatment. Several focus group participants expressed concern that if hepatitis was a serious illness, a general practitioner may not have sufficient knowledge and expertise to support patients undergoing hepatitis treatment.

### Capacity issues in general practice

General practitioners who worked in practices specifically for asylum-seekers and refugees reported that all patients who had moved to the UK from regions with high hepatitis B or C prevalence were typically screened in accordance with Health Protection Agency guidelines. Those in other practices reported that their antenatal patients from immigrant communities were routinely screened for hepatitis B and that contact tracing for family members of those who test positive was attempted. Screening for hepatitis C was perceived as more of a concern for patients who inject drugs, or when a patient showed abnormal liver function test results.

Some general practitioners expressed concern that establishing a new, independent hepatitis screening programme would be labour intensive and perhaps not the most effective use of limited practice resources at a time of increasing demands on the primary care sector. The prevailing policy of ‘care closer to home’ had meant that general practitioners were being asked to take on numerous tasks traditionally undertaken by hospital teams and hence were feeling under significant strain. While the convenience for patients of a primary care based hepatitis screening service was acknowledged, practices with limited physical space and without additional support services (such as phlebotomy), were felt unlikely to be capable of supporting such a service. Key informants and general practitioners highlighted the heavy time demands and challenges involved in following up immigrant patients for screening and treatment appointments, partly because of frequent changes of address within this population and high patient turnover.

Our sample of general practitioners reported that following a hepatitis diagnosis, they discuss the meaning of the diagnosis with the patient, including the implications for transmission and the process of referral to specialist hepatitis treatment services, and some played a role in the treatment journey of their patients after referral to specialist clinics (for example, providing information and emotional support, particularly when the patient felt unwell or distressed from the side-effects of hepatitis treatment). However, our sample was probably self-selecting for interest in hepatitis. Our key informant data suggested that many patients receive little information or support from their general practitioner following their diagnosis and only begin to learn about hepatitis when they attend specialist treatment services.

One general practitioner pointed out that hospital-based hepatitis clinics could provide multidisciplinary support to patients, including access to psychological support for patients undergoing treatment. Others suggested that general practitioners may lack knowledge and confidence in overseeing the active treatment of chronic viral hepatitis, or may not have the appointment capacity to monitor treatment. Some general practitioners expressed their frustration at the insufficient consideration of the long-term resource needs for an intervention to be continued following a positive pilot phase, even when positively evaluated and ‘evidence-based’.

## Discussion

### Summary of main findings

This qualitative study, the first of its kind in the UK, has surfaced a number of factors that must be taken account of when planning a comprehensive screening and treatment service for hepatitis B and C in high-risk immigrant communities. In particular, the level of awareness and knowledge of these conditions in immigrant communities was generally low, and at best variable; with misunderstandings about modes of transmission, limited awareness of the asymptomatic nature of chronic infection, and underestimation of the seriousness of hepatitis B and C infection. While a primary care based service for screening and treatment would be convenient for patients, language and accessibility barriers may remain an issue for immigrant communities. Our findings also raised questions both about patients’ confidence in general practitioners and the capacity of the primary care sector to deliver such a service. Table 3 provides a summary of the research findings, alongside their implications for the design of screening and treatment services.

**Table 3:** **Summary of main research findings and implications for design of screening and treatment services**

**Main findings**	**Implications**
***Limited knowledge and confusion about hepatitis B and C in at-risk communities***	***Community-based information campaigns***
• Participants reported a lack of awareness of the asymptomatic nature of chronic infection with hepatitis B/C within at-risk communities. Many immigrants may not consider themselves at risk from hepatitis B/C if they have lived in the UK for many years.	• Information is needed regarding the asymptomatic nature of chronic infection and the potentially serious outcomes of untreated infection.
• Chronic viral hepatitis does not typically feature in community discourses about serious illness, because many people are unaware of the outcomes from chronic infection, because those diagnosed often have no symptoms of illness, and because hepatitis has not received the same health promotion/ media coverage as other illnesses. People are typically more worried about illnesses such as cancer, diabetes and HIV.	• Information is needed to improve understanding of how hepatitis B and C may be acquired in high-risk regions and to amend misunderstandings about transmission.
• We found uncertainty and confusion about the differences between the types of viral hepatitis within at-risk communities. Many people indicated a belief that hepatitis C was more serious than hepatitis B.	• Collaborative working is needed between health educators and community groups, faith organisations, etc. to communicate verbal information about hepatitis and screening.
• There was some awareness within the focus groups about the main transmission routes of hepatitis B/C, but misperceptions were also reported that indicated confusion with the transmission of hepatitis A and other causes of liver disease.	
• High levels of stigma were generally not perceived for hepatitis, but stigma may arise due to perceived association with socially unacceptable behaviours, and due to fear of infection.	
***Barriers to hepatitis screening and treatment for immigrant patients***	***Service implications***
• PRACTICAL BARRIERS	• Information about screening and treatment provision ought to be provided in the languages of the communities that are targeted for screening. Language support services will be needed to assist patients with making and attending appointments.
- Language and communication difficulties are a major barrier for immigrant communities in accessing primary care.	• Flexible/extended opening hours may be needed for hepatitis screening and treatment services.
- The long working hours and limited working rights (e.g. no sick pay) of many immigrants were viewed as a significant barrier to accessing screening and treatment services.	• People need to be fully informed in advance about what is involved in the testing and treatment process, and that treatment is free of charge.
- Screening invitation letters may be ignored, particularly if the person does not understand hepatitis or does not perceive a need for screening.	• GPs or other primary care staff may need to verbally explain the reasons for hepatitis screening to the patient, rather than relying on screening invitation letters.
• PSYCHOLOGICAL BARRIERS	• Collaborative working with community groups to provide support to patients during treatment.
- Screening uptake may be prevented by fear of diagnosis, fear of the testing process involved and fear of potential side effects from treatment.	• Patients may need to be provided with reassurance and confidence that they will receive effective treatment for hepatitis through primary care services.
• Problems with trust and confidence in primary care amongst immigrant communities may reduce uptake of screening and treatment.	
***General practitioner concerns***	***Policy implications***
• Workload implications and concerns about sustainability may discourage general practice participation in the delivery of hepatitis screening and treatment services.	• Ongoing consultation with primary care services regarding support needs for delivery of hepatitis screening and treatment.

### Links to previous literature

Our findings confirm previous research on the confusion regarding the differences between the types of viral hepatitis [36], along with a folk model that the hepatitis A, B and C viruses are interconnected and progressive in their severity [22,37]. Hepatitis A is the most common form of viral hepatitis and has moderate to very high prevalence in the countries of origin of the communities in the current study [38], which may be why risk factors for hepatitis A have been incorporated into the folk model for hepatitis B and C. We also confirm findings in previous studies [22,37] that hepatitis is linked in folk models to ‘yellow’ or ‘jaundice’ and that many people are unaware that chronic hepatitis infection can be asymptomatic [36]. Similar to previous research [22,24,39], we found that hepatitis B and C are largely not viewed as serious, life-threatening illnesses within the target communities.

While we found some awareness of the key routes of transmission for hepatitis B and C, many people at high risk (by virtue of their country of origin) may be unlikely to perceive a need for hepatitis screening if they are unaware that hepatitis can be a chronic, asymptomatic infection that they may have acquired at a younger age. Absence of symptoms has been associated in previous studies with reluctance to attend for screening, [40] including for hepatitis [20]. Previous researchers have linked low public awareness of chronic viral hepatitis to the limited attention the condition has received in health education and media campaigns [36,41], and shown (as we did) that most awareness and knowledge of the condition comes from personal or family experience of the illness [42,43].

Our study found variation in the level of stigma associated with chronic viral hepatitis, with the greatest stigma attached to perceived associations between hepatitis and socially unacceptable behaviours, such as sexual promiscuity and injecting drug use; which may be reinforced by health promotion literature concerning the key modes of hepatitis B/C transmission in the white British population. Research by others has also shown that whilst stigma surrounding hepatitis is often lower than that for HIV [23,44,45], where stigma is high there is reluctance amongst patients to disclose a hepatitis diagnosis to others [46].

We found that immigrant communities in the UK may be fearful of day-to-day interaction (especially meals) with people who have hepatitis B or C infection, due to misperceptions regarding the transmission of these viruses. Perceived contagion risk has historically been associated with social and physical avoidance of individuals with disease [47] and fear of contagion has previously been shown to contribute to the stigma surrounding hepatitis B and C [43,45,46,48,49].

Doctor recommendation for hepatitis screening has previously been identified as strongly associated with screening uptake in immigrant communities [50-52]. We identified that there may be sensitivity amongst immigrant communities about being targeted ‘en masse’ for hepatitis screening, in contrast to a previous study of perceived acceptability of tuberculosis screening for immigrants that found little sensitivity to targeted screening in those who had been tested [53]. It is possible that people may find offence in a letter which points out that their community is at increased risk of an infectious disease, but welcome a detailed verbal explanation from their GP about the risks in their country of origin.

Health inequalities for minority ethnic populations are strongly associated with socioeconomic disadvantage; [54] though utilisation of healthcare services, including screening uptake, can vary by ethnicity [55,56]. Our findings corroborate previous work showing that structural and practical barriers (notably the stresses and constraints of socio-economic disadvantage, and low health and system literacy) have an important role in access to optional screening services [33]. Uptake of national screening programmes is generally lower amongst people from socially disadvantaged areas [57]. The more rigid working patterns involved in manual employment have previously been associated with greater perceived difficulty in accessing healthcare, particularly for optional, preventive health services [21,58]. These are likely to affect various immigrant communities in different ways, given the very different employment patterns by ethnic group [59].

Disadvantaged groups are more likely to have a negative outlook on future events and to experience increased feelings of threat, which means that they are more likely to expect testing procedures to be unpleasant and uncomfortable, and to worry about the outcomes of screening, and the physical and psychological consequences of a diagnosis [33]. As in our study, fear of acquiring a frightening diagnosis has been found to be an important barrier to screening uptake in minority ethnic communities [60].

Language and literacy were consistently identified as important influences on the willingness and ability of immigrants to engage with hepatitis screening and treatment programmes. Language and communication difficulties are amongst the strongest influences on help-seeking behaviour, access to healthcare, and experiences of services for minority ethnic communities [55,61,62], and a bigger barrier to screening engagement than attitudes to screening [63]. Reliance on family members to translate can constrain availability to attend services and compromise the accuracy and quality of information exchanged [64], though the use of trusted family members can in some circumstances improve access for those who perceive the official interpreting services as unsympathetic or alienating [65], or who cannot otherwise access interpretive services.

Our finding that uptake of a primary care based screening and treatment service is likely to depend on the strength and quality of the GP-patient relationship (which in turn depends on continuity of care and the perceived time available for dealing with patients’ own priorities) resonates strongly with previous research on the key determinants of trust and confidence in GP care [66-69], particularly for minority ethnic patients [70]. Trust, satisfaction and continuity of care have been found to influence receptiveness to doctor recommendations about preventive care and screening intentions [71,72].

Our findings indicate that some people within the target communities may have limited confidence in a general practice-based service to manage chronic viral hepatitis, if they are aware of its seriousness. The more serious an illness is perceived to be, the more likely a patient will want a referral to a specialist and the less likely they will be to trust their GP’s management of the illness [73]. Recent immigrants from a country with a poor or absent primary health care sector may be particularly distrustful of GPs and associate ‘quality’ care with hospital referral, extensive investigations and prescribed medication [66,67].

This study indicated that even GPs with an interest in hepatitis management were concerned about their capacity to support a screening and treatment service. Previous studies have revealed similar concerns amongst GPs about both the resource burden of transferring services from secondary to primary care [73], and the time and resource challenges to being involved with the delivery of healthcare interventions for research [74-76].

### Strengths and limitations of the study

By exploring how hepatitis B and C are viewed in high-risk immigrant communities and the perceived acceptability of a primary-care model of screening and treatment, this study makes an important contribution to the development of strategies to test and treat population groups at greatest risk of chronic viral hepatitis. The validity of our findings is strengthened by our multi-phased approach to data collection, including the perspectives of members of communities at increased risk, key people working within those communities, specialist healthcare professionals working in hepatitis treatment services, and general practitioners. Previous studies of knowledge and attitudes towards viral hepatitis in immigrant communities have focused on either hepatitis B or C, and have typically limited their data collection to one particular immigrant group (mostly Asian communities in North America). To gain a more comprehensive picture, the current study looked at understanding of both conditions across several high-risk communities and is the first study to include perspectives on hepatitis B and C amongst Roma, Somali and other African communities. We have illustrated that many of the barriers to hepatitis screening (including low knowledge about hepatitis B and C) are shared across the target communities, a similar finding to previous research on barriers to cancer screening in minority ethnic groups [57]. Whilst we found differences in understanding and folk models of viral hepatitis between the community groups, this study was specifically oriented to informing the design of a standardised intervention to be rolled out in a multi-ethnic community locally (and, we anticipate, nationally). Furthermore, our sample was too small to draw confident conclusions about inter-group differences. Future research targeted at particular communities could further explore culturally-specific influences on hepatitis screening behaviour.

Focus group participants were recruited through community organisations which may have caused selection bias [23]. Those recruited were generally known to the organisations, thus were more likely to be people with relatively high health literacy who actively engage in community health events. Through our research design we aimed to circumvent the potential selection bias of the focus group sample. By interviewing key informants who worked in a range of different roles within the communities under study, we gathered an insight into the spectrum of attitudes to hepatitis and the range of healthcare barriers faced by members of these communities, including those less likely to participate in research. Furthermore, the use of vignette techniques in the focus groups encouraged participants to speak about factors affecting people in their wider community which may influence engagement with hepatitis screening, in addition to their own personal intentions. However, future research ought to widen data collection within each immigrant community to include the views of community members who are perhaps more isolated from health and community support services, and whose perspectives and experiences may differ from those that are represented in our focus group data. Snowball sampling could perhaps be used, where people known to organisations could be asked to inform other friends and relatives who are not linked in with services about the research.

There was a wide age range in our focus groups. We did not stratify by age, which may have affected the findings. For example, younger Roma women contributed relatively little to group discussions, perhaps because of a cultural tendency to defer to more senior members. Our data suggested that younger people within immigrant communities were perceived to have greater access to health information and to be more open to testing for sexually transmitted infections than older ones, but may be less likely to perceive a need for screening if they feel healthy. Future research could explore these issues in greater depth using stratification by age or generation.

A key limitation of the current study is that we cannot extrapolate participants’ responses to a hypothetical screening invitation, since intentions to attend screening are typically higher than actual attendance [77]. Given the well-described gap between what people say they would do and what they actually do, future research should explore the ‘real world’ experiences and health needs of immigrant groups who are offered screening and treatment for hepatitis B and C.

There were also limitations with our GP data collection. A small number of GPs were willing to be interviewed; some of those who did not participate indicated that they already had too many constraints on their time. The representativeness of our GP informant sample therefore requires consideration, thought the current study does not claim that our findings regarding the capacity issues that may affect a primary care-based intervention for chronic viral hepatitis are representative of all GP practices that may be approached to deliver such an intervention. Further qualitative research at a later point in the *‘HepFree*’ study with primary care practices who do participate in delivering the intervention may uncover different GP perspectives on negotiating the time and resource demands of research. There is also likely to have been a selection bias in the sample of general practitioners interviewed for this study, who reported that they actively support their patients undergoing hepatitis treatment. Our key informant data provided a wider perspective on GP engagement with chronic viral hepatitis services, which included those who gave less priority to the condition. We also acknowledge that asking GPs about a single question covering their approach to hepatitis B and C may have reduced our potential to explore differences in their management of these two different diseases.

## Conclusions

This paper has identified a range of issues likely to affect engagement with hepatitis screening and treatment in primary care for immigrant communities. The concept of ‘candidacy’ proposed by Dixon-Woods et al. [55] as a means of understanding access to healthcare services by vulnerable groups is a useful framework for interpreting these findings. They outline how *“people’s eligibility for medical attention and intervention is jointly negotiated between individuals and health services” (pp.42)*. In the case of a screening invitation, candidacy, or eligibility for screening is proposed by the healthcare provider. In order for a person to then present themselves for screening, they must accept that the provider statement of their eligibility is consistent with their own perceptions of eligibility, and that the invitation is worth acting upon in terms of the resources and efforts that are needed in order to engage with the screening service.

With regards to hepatitis screening, response to a screening invitation from the GP will firstly depend on whether the patient perceives themselves to be at risk of chronic hepatitis infection, which is likely to be influenced by their awareness and understanding of the condition. Secondly, their response will depend on the challenges that they may face in mobilising the psychological and practical resources to navigate the screening and treatment process. While a community-based model of screening and treatment in primary care was largely viewed as a positive development in the current study, numerous challenges were outlined which immigrant communities may face in engaging with such a model; including fear of diagnosis, language and communication difficulties, work demands, and issues with trust and confidence in GP care. The magnitude of these challenges is likely to vary both within and across communities; for example, engaging with healthcare services may be particularly difficult for immigrants who are living in the UK without family support, or without people to communicate on their behalf with healthcare services.

### Implications for screening and treatment services

The *‘HepFree’* study (funded by the National Institute for Health Research) will examine the clinical and cost-effectiveness of a large programme of hepatitis screening and treatment in NHS primary care services, with the aim to provide evidence-based recommendations to the National Screening Committee. The research presented in this paper has identified contextual factors relating to expected acceptability and engagement with such a programme in high-risk immigrant communities, thus highlighting key issues which are likely to affect the design, implementation and outcomes of the *‘HepFree’* study. The implications of our research finding for the delivery of hepatitis screening and treatment services in primary care are summarised in Table 3.

Our findings indicate that information campaigns are needed to address limited understanding of chronic viral hepatitis within at-risk immigrant communities. In particular, information is needed regarding how hepatitis B and C are transmitted in high-risk regions, the asymptomatic nature of chronic infection and the outcomes of untreated chronic infection. At the same time, information campaigns must take care not to cause alarm or fear about these infections, which may discourage screening attendance through fear of diagnosis, and may exacerbate any stigma for people with a diagnosis of hepatitis B or C. Information that amends misunderstandings about hepatitis B and C transmission may also reduce potential stigma. Information about hepatitis and encouragement for screening may need to be communicated verbally through community and religious organisations, and detailed explanations from GPs or other practice staff, in addition to initial screening invitation letters. Verbal explanations and support for screening may reduce patient fears surrounding the testing process and the potential outcomes of a diagnosis.

To reduce language and communication barriers to hepatitis screening and treatment, services will need to provided information (written and verbal) in the languages of the communities that are targeted, and the availability of interpretive services to assist people with making and attending appointments must also be considered. Services may need to be flexible in terms of their opening hours to accommodate the inflexible working hours of many immigrant patients. If community-based treatment for chronic viral hepatitis is to be provided in primary care services, patients may need to be provided with confidence and reassurance that effective treatment can be provided in primary care. Primary care practices will also need to receive ongoing support and consultation in relation to their capacity for delivering such a model.

## Appendix 1: Key informant interview questions

General

- What do you think are the key health issues affecting the X community in London?
- What do you think are the key issues that affect this community in terms of accessing GPs and health services?

Hepatitis

- From your experience, what do people think about hepatitis B and C within this community?
- How do they think hepatitis B and C are transmitted?
- Do you think people within the X community think that they are at risk for hepatitis?
- How do people hear about hepatitis within this community?
  - Who do people talk to about hepatitis?

Screening

- What do you think are the reasons why people from the community get tested for hepatitis?
  - What do you think are the reasons why people do not get tested?
  - How do you think things could be improved to support people to get tested for hepatitis?

### Explain planned intervention of GP-based screening and treatment for chronic viral hepatitis

- How do you think people will react to a hepatitis screening invitation letter from their GP?
- What kind of challenges do you think there might be in recruiting people for testing in this way?
- Do you think there are any sensitive matters we will need to pay attention to in sending these letters out to the X community?
- Are there any cultural factors which are specific to the X community that you think may have a role in whether or not people get tested for viral hepatitis?

Treatment

- Do people in the community tend to engage with treatment for hepatitis if diagnosed?
- Why do you think some people who test positive for hepatitis may not seek treatment?
- Do people seek care and treatment from any other sources (alternative medicine)?
- How do you think people from the community could be best supported to get treatment for hepatitis?
- What kind of effects does a hepatitis diagnosis have for people in the X community?

## Appendix 2: Focus group topic guide/key discussion questions

- What do you think are important health issues for the X community living in London?
- Do you know of any infections or diseases that affect the liver?
- Can anyone explain what hepatitis B and C are?
- Has anyone ever talked about hepatitis with family or friends?
- Does anyone know how people get hepatitis B or C?
- Do you think the X community is at risk for hepatitis?
- How would someone know if they had hepatitis B or C?
  - Information piece about hepatitis B and C presented verbally to group
  - Vignette presented verbally to group: “This [silhouette image] is a woman, Mrs. N-, from the X community living in London. She has just received a letter from her GP inviting her to be tested for viral hepatitis.”

- What will she be thinking when she gets the letter?
- Why do you think she might not want to get tested?
  - What might she be worried about?
  - What would make it difficult for her to go for testing?

- Why do you think she might decide to go for testing?
- If she asked her family or friends for advice about whether to go for the test, what do you think they would say?
- If your friend came to you for advice about whether to go for the test what would you say?
- Who could she go to for more information about hepatitis?
- If she knew how serious viral hepatitis is do you think she would be tested for it?
- Do you think there would be any differences between a man and a woman about whether they would want to get a test for hepatitis?
- If we wanted to encourage lots of people from the X community in London like Mrs. N- to come for testing, what should we say in an invitation to them?

### Vignette continues: “Mrs. N- has been tested and the doctor has told her that she has chronic viral hepatitis”

- What would she think when she discovers this?
- What do you think her family and friends will think about this? What will other people in the community think?
- Do you think it would affect her life in any way?
- Where do you think she would go for treatment?

### Vignette continues: “Mrs. N-’s GP has told her that she can receive treatment for her hepatitis at the GP service”

- Do you think she would want to receive treatment for hepatitis from her GP service? (Why/why not?)
- Is there anything that might make it difficult for her to get treatment?

## Appendix 3: General practitioner interview questions

- Who do you typically test for hepatitis B and C at your practice?
- Can you recall particular situations in which the issue of testing for hepatitis has arisen with your patients, and how this was discussed?
- How is a positive result for hepatitis managed in your practice?
- Do you discuss the treatment plan, etc.?
- What do patients typically need from you following a hepatitis diagnosis?
- Do you think many GPs offer hepatitis testing to their patients from ethnic minority or immigrant communities? (Why/Why not?)
- What kind of barriers do you think there are for GPs in offering hepatitis tests to their patients from ethnic minority or immigrant communities?

### Explain planned intervention of general practice-based screening and treatment for chronic viral hepatitis

- What kind of reaction do you think your patients would have to a hepatitis screening invitation letter?
- What do you think about providing treatment for hepatitis at GP practices?
- What kind of benefits/ problems do you think there might be with this kind of intervention?
